# Calciphylaxis Following Parathyroidectomy in Chronic Kidney Disease Patients—Case Report and Literature Review

**DOI:** 10.3390/biomedicines13030715

**Published:** 2025-03-14

**Authors:** Nada Akad, Stefana Catalina Bilha, Mugurel Apetrii, Fawzy Akad, Madalina Bilha, Mihai Hogas, Simona Hogas, Maria-Christina Ungureanu, Cristina Preda, Adrian Covic

**Affiliations:** 1 Nephrology Department, “Grigore T. Popa” University of Medicine and Pharmacy, 700115 Iasi, Romaniaaccovic@gmail.com (A.C.); 2 Endocrinology Department, “Grigore T. Popa” University of Medicine and Pharmacy, 700115 Iasi, Romania; 3 Anatomy Department, “Grigore T. Popa” University of Medicine and Pharmacy, 700115 Iasi, Romania; 4 Pathology Department, “Grigore T. Popa” University of Medicine and Pharmacy, 700115 Iasi, Romania; 5 Physiology Department, “Grigore T. Popa” University of Medicine and Pharmacy, 700115 Iasi, Romania

**Keywords:** calciphylaxis, end-stage renal disease, vascular calcification, parathyroidectomy, calcium

## Abstract

Calcific uremic arteriolopathy, also known as calciphylaxis, is a rare and often fatal condition most commonly occurring in patients with end-stage renal disease (ESRD). It is marked by extensive vascular calcification, resulting in tissue ischemia and the development of distinctive skin lesions. We report the case of a 38-year-old male with ESRD due to polycystic kidney disease, who developed calciphylaxis lesions following total parathyroidectomy (PTx). We also performed an electronic search of PubMed and Google Scholar from inception until December 2024, using the following keywords: ‘chronic kidney disease’, ‘dialysis’, ‘calciphylaxis’, ‘calcific uremic arteriolopathy’, ‘secondary hyperparathyroidism’, and ‘parathyroidectomy’. A literature review of calciphylaxis cases following PTx in chronic kidney disease (CKD) patients identified 14 cases reported up to the manuscript’s writing. Although PTx can be a treatment option for calciphylaxis related to severe secondary hyperparathyroidism (SHPT), leading to clinical improvement in some patients, there are atypical calciphylaxis cases occurring after PTx. While the mechanism is not fully understood, the sudden reduction in parathormone (PTH) levels leading to hypocalcemia and decreased bone turnover, together with an increased calcium loading in a patient at risk for abnormal mineralization, may promote vascular and soft tissue calcification. However, the long-term impact of severe SHPT with a delayed post-PTx manifestation cannot be ruled out. Clinicians should consider calciphylaxis in CKD patients with new painful skin lesions. Skin biopsy remains controversial, but a thorough clinical examination, and, in some cases, imaging are essential for a correct diagnosis. A multidisciplinary, personalized approach is crucial, with careful management of post-PTx hypocalcemia and calcium supplementation. Further research is needed to enhance understanding and treatment strategies.

## 1. Introduction

Calciphylaxis, also referred to as calcific uremic arteriopathy (CUA), presents as a rare yet profoundly impactful disorder characterized by vascular calcification. Its primary manifestations include calcification within the subcutaneous adipose tissue and small blood vessels of the dermis. This calcification is often accompanied by intimal fibrosis and microthrombosis, ultimately leading to tissue ischemia, necrosis, and the development of skin ulcers [1]. It mainly occurs in individuals with chronic kidney disease (CKD) undergoing dialysis (uremic calciphylaxis), but cases have also been reported in patients with earlier stages of CKD, in kidney transplant recipients (KTR) [2] and in subjects with normal renal function (non-uremic calciphylaxis) [3]. The occurrence of CUA in dialysis patients varies from 1 to 4%, with an annual occurrence rate of approximately 0.04% and the annual mortality rate is documented to be as high as 80% among patients with CUA [1,4]. Ulcerated lesions are linked with higher mortality rates compared to non-ulcerated lesions, with sepsis identified as the primary cause of death [5,6]. In hemodialysis (HD) patients, mortality rates among those with calciphylaxis were nearly three times higher than among those without calciphylaxis, according to data from the United States Renal Data System [7]. The prognosis for this disease is poor, and while various diagnostic schemes have been suggested, there is presently no universally accepted standard. Post-parathyroidectomy (PTx) calciphylaxis is rare and atypical, and its mechanism is not fully understood. It has been hypothesized that it may result from decreased bone turnover due to a sudden drop in parathyroid hormone (PTH), leading to an elevated circulating pool of calcium (Ca) and phosphate (P), which promote vascular deposition and calcification [8]. High calcium loading, leading to a high Ca × P product is a well-recognized risk factor for calciphylaxis [9]. Adynamic bone disease, which is usually associated with a low PTH level, is also known to favor ectopic calcification [10]. Further on, low levels of vascular calcification inhibitors, such as fetuin-A which prevents calcium and phosphate deposition, are low in HD patients [11,12]. More so, high levels of inactive, uncarboxylated matrix Gla protein (MGP) which are indicative of low vitamin K status in HD patients, are associated with higher calcification scores [12]. Thus, a decreased calcium and phosphate uptake by bone due to sudden drop in PTH together with an impaired serum clearance, may lead to vascular deposition and calcification [13]. We report a case of calciphylaxis lesions occurring post-PTx in a young male with stage 5 CKD undergoing chronic HD. Literature data reporting post-PTx calciphylaxis in CKD patients were also reviewed and diagnosis, risk factors contributing to calciphylaxis events and the available therapeutic approaches are discussed.

## 2. Case Report

**Figure 1:**
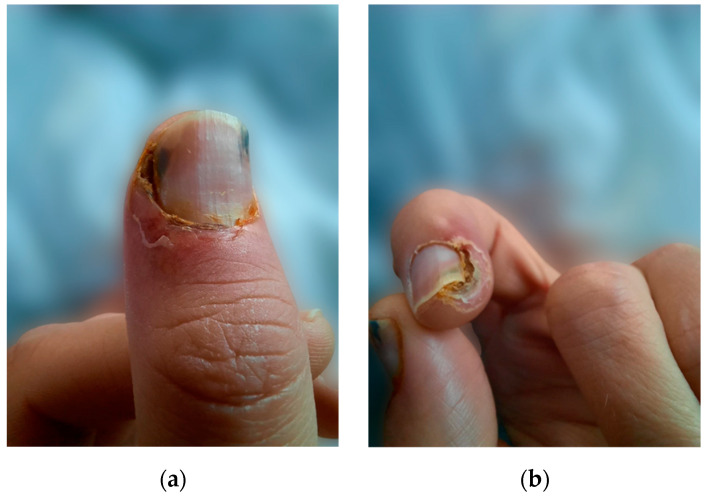
(**a**) Calciphylaxis lesions on the thumb, (**b**) calciphylaxis lesions on the index.

We present the case of a 38-year-old male undergoing HD with a history of end-stage renal disease (ESRD) secondary to autosomal dominant polycystic kidney disease on renal replacement therapy (RRT) for the last 18 years. Other past medical history included arterial hypertension, secondary osteoporosis due to secondary hyperparathyroidism, and virus C hepatitis. At 35 years of age, due to markedly elevated PTH levels reaching 2100 pg/mL (normal range in general population 15–65 pg/mL) despite optimal medical treatment, he underwent total PTx, resulting in an initial reduction of PTH to 330 pg/mL and the onset of severe hypocalcemia. Following the administration of calcium supplements and vitamin D analogs, the patient developed painful necrotic plaques on the fingers, which were subsequently diagnosed as classic calciphylaxis lesions (Figure 1). After a thorough evaluation of clinical features and underlying comorbidities, differential diagnosis was made with livedo reticularis, cholesterol embolism, pyoderma gangrenosum, and vasculitis. Consequently, it was decided to postpone calcium and vitamin D supplementation and a skin biopsy was considered. However, the spontaneous resolution of ischemic skin lesions after discontinuing calcium supplements in the absence of inflammatory syndrome, anticoagulation or eosinophilia supported the diagnosis of CUA and led to reconsideration of the need for a skin biopsy.

**Figure 2:**
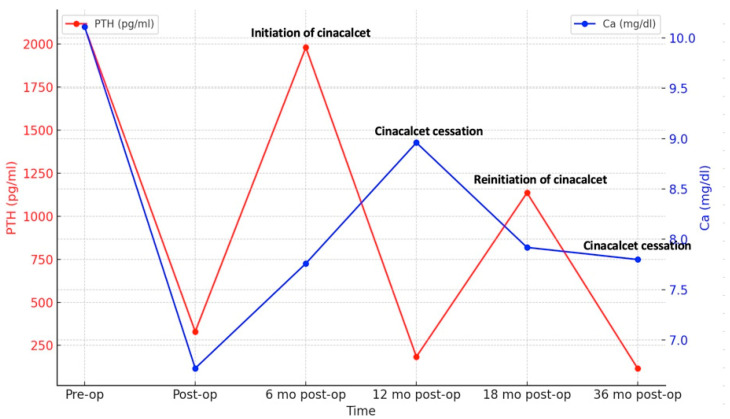
Dynamic changes in serum PTH and calcium.

Six months after surgery, PTH levels began to rise again, peaking at 1980 pg/mL (Figure 2) and prompting initiation of cinacalcet 30 mg/day. Despite the patient experiencing hypocalcemia at the time, correction with calcium and vitamin D analogs was postponed due to the risk of CUA recurrence, especially as he was asymptomatic. Serum PTH and calcium levels fluctuated over time (Figure 2), leading to discontinuation of cinacalcet 12 months postoperatively when PTH had dropped to 184 pg/mL, below the target values for dialysis (the normal upper limit is 2–9 according to CKD-Mineral Bone Disorder (CKD-MBD) guidelines [14]). Cinacalcet was reinitiated 18 months postoperatively and definitively discontinued 36 months postoperatively when serum PTH fell again to 142 pg/mL (below target range). Dual X-ray absorptiometry (DXA) performed one year before surgery and repeated 2 years later revealed improved bone mineral density at the femoral neck, with persistent radius low Z-scores (Table 1).

**Figure 3:**
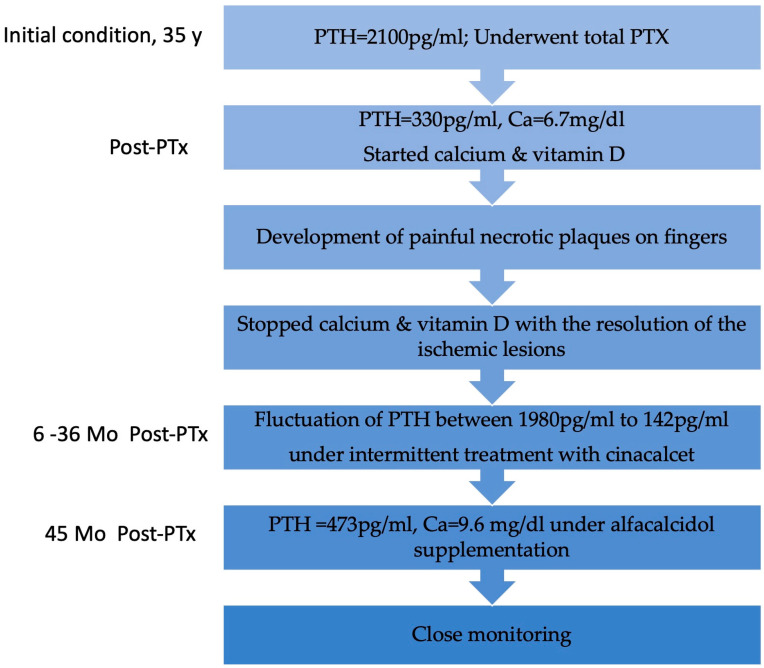
Flowchart with the patient’s clinical evolution. Y, year; PTx, parathyroidectomy; Mo, months.

Follow-up visits after definitive discontinuation of cinacalcet showed a progressive rise in serum PTH levels, which remained within the target range. Despite experiencing hypocalcemia both before and during cinacalcet treatment, he remained asymptomatic throughout. An improvement in serum calcium levels was observed following the initiation of alfacalcidol 0.5 mcg/day. Nine months after stopping cinacalcet therapy, PTH levels increased to 473.4 pg/mL and corrected calcium level was 9.6 mg/dL, indicating adequate control of calcium metabolism, though close monitoring remained necessary. No recurrence of ischemic lesions was observed during follow-up. The patient’s clinical course is illustrated in Figure 3.

## 3. Literature Review

Calciphylaxis is a concerning complication in ESRD patients on RRT, necessitating comprehensive interdisciplinary management. Its occurrence following PTx is extremely rare, as evidenced by the limited number of documented cases in the literature. We conducted electronic searches using PubMed and Google Scholar, from inception to December 2024, employing combinations of the keywords ‘chronic kidney disease’, ‘dialysis’, ‘calciphylaxis’, ‘calcific uremic arteriolopathy’, ‘secondary hyperparathyroidism’, and ‘parathyroidectomy’. We systematically reviewed the retrieved literature and pertinent references cited within, limiting our review to articles written in English. Table 2 provides a summary of all reported cases to date [2,8,13,15,16,17,18,19,20,21,22].

We identified 14 other cases reported in the literature, 13 patients undergoing HD and one KTR. Including our case, the majority of these cases (9 out of 15) involved male patients, with a mean age of 47.8 years. The calciphylaxis appeared after a mean dialysis vintage of 11.1 years in HD patients. Only one case [18] did not mention the dialysis vintage period. In most cases, PTx was performed due to secondary hyperparathyroidism (SHPT). However, two cases [18,19] underwent the procedure because of prior calciphylaxis events and one case mentioned the presence of a brown tumor [8]. Nine out of the 15 cases had subtotal parathyroidectomy. The preoperative PTH values were greater than 1205 pg/mL in HD patients, with values reaching up to 4000 pg/mL. PTx was followed by a rapid decline in PTH levels, with postoperative PTH values between 9 pg/mL and 705 pg/mL. Calciphylaxis events occurred between 2 weeks and 3 years post-PTx. In most of the cases (9/15), skin biopsy confirmed the diagnosis. The KTR patient needed limb amputation, so diagnosis was made on the histopathology of the affected area [2]. Other diagnostic methods used included X-ray, 99mTc-methylene disphosphonate, and computed tomography scan [16].

## 4. Discussion

Calciphylaxis, characterized by intricate vascular calcification and a complex pathogenesis, results in severe vascular compromise due to thrombotic occlusion within blood vessels, accompanied by calcification along their inner lining. It manifests through intensely painful skin lesions, including livedo reticularis, reticulate purpura, indurated nodules, and violaceous plaques. These lesions frequently undergo complications such as blister formation, ulceration, necrosis, and secondary infections [23]. Uremia, calcium-derived compounds, and reactive oxygen species associated with ESRD are believed to contribute to vascular calcium deposition and fibrosis, ultimately leading to the development of calciphylaxis [24]. In a retrospective study involving 36 patients, spectroscopy revealed that vascular wall depositions in calciphylaxis consist solely of calcium apatite, organized circumferentially within small and medium-sized arteries. This suggests a pathophysiology different from atherosclerosis [25]. Microvascular calcification is the primary mechanism driving the condition, but adipocyte proinflammatory cytokine signaling and vascular endothelial injury also contribute [6]. Calciphylaxis can be classified into central and peripheral types based on the location. The central type, common in obese patients, affects areas abundant in subcutaneous fat tissue, such as the abdomen, buttocks, thighs, or breasts. The peripheral type affects areas with less adipose tissue, such as the hands, feet, and penis, and is characterized by severe pain [1].

**Figure 4:**
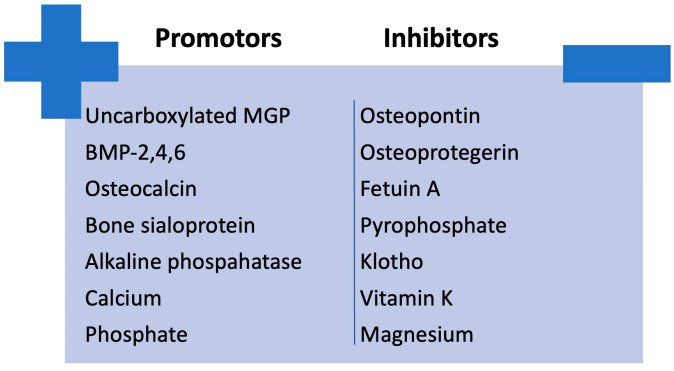
Imbalance between calcification promoters and inhibitors in CKD. BMP, bone morphogenetic protein; MGP, Matrix Gla protein.

An imbalance between factors that promote calcification (e.g., bone morphogenetic protein (BMP)-2, 4, and 6; osteocalcin; bone sialoprotein; alkaline phosphatase; calcium; phosphate) and those that inhibit it (e.g., carboxylated MGP, osteopontin, osteoprotegerin, fetuin A, pyrophosphate, klotho, vitamin K and magnesium) in CKD could lead to the development of vascular calcification [26,27]. The imbalance between calcification promoters and inhibitors is illustrated in Figure 4.

MGP, released by chondrocytes and smooth muscle cells in blood vessels, inhibits vascular calcification, but requires phosphorylation and vitamin K-dependent carboxylation to function. A study conducted on 112 patients demonstrated a deficiency of vitamin K in hemodialysis patients and a higher uncarboxylated MGP [26]. This prompts questions about the potential advantages of administering vitamin K supplements to patients with CKD.

In impaired kidney function, reduced vitamin D synthesis and compromised calcium and phosphate regulation lead to secondary hyperparathyroidism, which can cause bone remodeling and arteriolar microcalcification [6]. Calcium levels can vary in CKD. In the evolution of the kidney disease, hypocalcemia usually occurs first due to reduced 1,25-dihydroxyvitamin D levels, leading to impaired calcium absorption and decreased active calcium transport [28]. Calcium levels may normalize or even increase during the course of advanced CKD, often due to excessive intake of calcium-based phosphate binders or vitamin D analogs which enhance calcium absorption or due to SHPT [14]. Nevertheless, dialysis patients may still be at risk of developing calciphylaxis despite serum calcium and phosphate levels within the normal range [6]. Regarding vitamin D, there have been studies showing the association of vitamin D receptor polymorphisms with a higher probability of CUA [29].

Several risk factors can contribute to the development of calciphylaxis. Major risk factors include dialysis vintage of more than 5 years, prolonged usage of warfarin, long-term use of high-dose calcium-phosphate binders, an active vitamin D dose exceeding 0.5 μg/d, deficiencies in plasma protein C or protein S, deficiency in vitamin K, and intact PTH (iPTH) levels surpassing 1000 pg/mL. Meanwhile, dialysis for less than 5 years, obesity, diabetes mellitus (DM), hypoalbuminemia, prolonged use of immunosuppressants and glucocorticoids, hypercalcemia, hyperphosphatemia, iPTH levels below 300 pg/mL, subcutaneous injection of insulin or heparin, and iron overload were considered minor factors. Another factor worth mentioning is the association of autoimmune conditions such as rheumatoid arthritis, anti-phospholipid antibody syndrome, temporal arteritis and systemic lupus erythematosus raising the possibility of a potential role for autoimmunity in CUA development [1,2,7].

In the previously published cases, calcium supplementation for post-PTx hypocalcemia was involved in five cases [2,15,19,20], and vitamin D analogs were used in four cases [2,19,20] including our case. This finding emphasizes that correction of hypocalcemia acts as an exacerbating factor, as the administration of calcium supplements may contribute to the development or worsen the progression of calciphylaxis lesions. DM was a contributing factor in two instances. Topical and systemic glucocorticoids were involved in two cases [15,21]. The systemic glucocorticoids were administered for pain relief when no other effective methods were available. Anticoagulation was noted in one HD case, but the specific agent was not mentioned, and in the KTR case, where treatment with warfarin for atrial fibrillation was noted [2,18]. Although anti-vitamin K anticoagulants are linked to the worsening of calciphylaxis, direct oral anticoagulants seem to be safer and better tolerated [30].

Diagnosing calciphylaxis is challenging. In kidney disease patients, painful nodules, indurated plaques, dusky livedoid plaques, and/or non-blanching retiform purpura should raise suspicion [6]. The necessity of skin biopsy for diagnosing calciphylaxis in chronic dialysis patients remains controversial, balancing the risks of biopsy-related complications against the need to distinguish calciphylaxis from other conditions with differing treatments [31]. Calciphylaxis is primarily diagnosed clinically; however, a biopsy may be warranted to exclude other similar conditions. ESRD patients are less prone to initial misdiagnosis, as healthcare providers often consider calciphylaxis as a differential diagnosis [6]. Nevertheless, Musso et al. [31] proposed searching for specific “clinical pearls” that would rule out calciphylaxis when in doubt, such as purulent ulceration with undermined borders (pyoderma gangrenosum), ochre dermatitis (venous ulcer), infectious cellulitis, sclerotic skin induration (systemic nephrogenic fibrosis), distal lesions under the knee in atherosclerotic ischemic ulcer, and livedo reticularis, gangrene or cyanosis (atheroembolism). In our case, a skin biopsy was not performed as the lesions had typical appearance and began to improve after discontinuing calcium and vitamin D supplements. Other authors have reported the need for surgical debridement [22], or even more radical surgery with mastectomy being performed after breast skin biopsy due to lack of healing and severe pain [18]. Further imaging techniques including radiographs, computed tomography scans, ultrasonography, nuclear bone scintigraphy, and mammograms have generally been employed to assess calciphylaxis. Fetuin A levels have been used in trying to aid in the diagnosis of CUA. Nevertheless, systematic evaluations of these tools have not been conducted, and they are not currently endorsed for clinical utilization [5,6,23].

The management of CUA includes wound care and symptomatic treatment, aiming to limit necrotic tissues and prevent infection, as sepsis is the primary cause of mortality in these patients. Hyperbaric oxygen therapy and sterile maggot therapy are options for wound care, but limited data are available [5].

**Figure 5:**
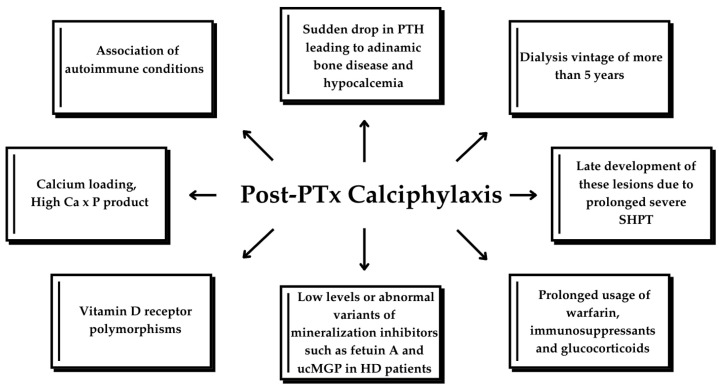
Factors leading to calciphylaxis in CKD patients. ucMGP, uncarboxylated matrix G1a protein; HD, hemodialysis; SHPT, secondary hyperparathyroidism.

PTx is an effective treatment option for calciphylaxis; however, it is not a definitive treatment, as calciphylaxis can still occur, though rarely, even after PTx. Low PTH levels alone are a risk factor for calciphylaxis [23]. PTx is believed to improve calciphylaxis-related outcomes by correcting the abnormal calcium–phosphate–PTH metabolism, as evidenced by previous cohorts and case reports. However, recent studies suggest that PTx may not be suitable for all calciphylaxis patients, particularly those at risk for postoperative complications. Furthermore, recurrent or de novo calciphylaxis has been reported following surgical PTx [32], as reported in the cases above (Table 2). Successful PTx often translates into reduced bone turnover and mineralization and severe hypocalcemia due to sudden drop in serum PTH [33]. This diminished calcium uptake by bones, together with calcium loading to correct hypocalcemia in a dialysis patient with reduced circulating levels or abnormal variants of mineralization inhibitors, such as Fetuin A or uncarboxylated MGP, are thought to lead to the accumulation of excessive calcium and phosphate deposition in vessels’ walls, particularly within the tunica media and soft tissues [9,21]; however, the exact mechanism is not fully understood. Nevertheless, PTH decreased dramatically after PTx in most cases and some authors reported calcium and vitamin D analog administration for correcting hypocalcemia, similar to our case [2,15,19,20,21]. Finally, the late development of these lesions due to prolonged severe SHPT with unknown trigger factors occurring after PTx cannot be ruled out. Figure 5 illustrates the pathophysiological mechanisms underlying the development of calciphylaxis in CKD patients.

One vital aspect is to address and modify risk factors such as abnormalities in the CKD-MBD axis. Maintaining serum calcium and phosphorus levels within normal ranges is essential. The optimal PTH level in calciphylaxis remains uncertain, but extremes of both high and low values should be avoided [5,21]. Vitamin D analogues such as calcifediol and paricalcitol, together with phosphate binders, are considered first-line therapy for optimizing biochemical abnormalities in CKD-MBD. Further on, calcimimetic drugs such as cinacalcet significantly reduce PTH levels, and may further help in controlling SHPT [34]. Cinacalcet is associated with an 83.4% overall response rate (complete or partial) and a 41.7% complete response rate when used in monotherapy in SHPT patients with calciphylaxis [35]. Newer calcimimetic agents include etelcalcetide, which achieves a 10% greater reduction in PTH levels compared to cinacalcet but does not improve gastrointestinal tolerance and carries a higher risk of hypocalcemia. Evocalcet, developed to address the limitations of cinacalcet, exhibits enhanced bioavailability, reduced CYP2D6 inhibition, and a lower incidence of gastrointestinal adverse effects. Upacicalcet, a novel non-peptidic injectable calcimimetic, demonstrates increased clearance via HD and does not impact gastric emptying [36].

PTx is preferred for treating SHPT in calciphylaxis patients with hypercalcemia and/or hyperphosphatemia. Calcium-based phosphate binders should be avoided due to the risk of exacerbating hypercalcemia through increased calcium absorption [37]. However, high-surgical risk patients or patients developing calciphylaxis post-PTX remain a challenge.

HD optimization to achieve the National Kidney Foundation-Kidney Disease Outcomes Quality Initiative (NKF-KDOQI) goals of dialysis adequacy may require an increase in frequency to four to five sessions per week to achieve sufficient phosphate removal. However, there are no confirmatory data that routine intensification of dialysis in patients with optimal adequacy parameters improves outcomes in patients with calciphylaxis [6].

Regarding pharmacotherapeutic agents, sodium thiosulfate (STS), first reported for successful use in 2004, is now widely recognized as a standard therapy for calciphylaxis. Multiple mechanisms have been proposed to explain its therapeutic benefits. Acting as a calcium chelator, sodium thiosulfate has been observed to decalcify calcified vessel walls. Thiosulfate binds calcium ions, forming highly water-soluble complexes that are eliminated via urine or dialysis. Additionally, as an antioxidant, sodium thiosulfate may counteract reactive oxygen species, thus mitigating inflammation, thrombosis, and vasoconstriction [38]. It is typically administered intravenously during the final hour of a hemodialysis session, the treatment period taking 3–6 months. It can also be injected around the border of the lesion or into the center of the lesion [38,39].

Evidence supporting the use of STS primarily comes from retrospective studies and case series. However, meta-analyses of data from these retrospective studies have found no significant differences in the risk of mortality, wound progression, or amputation between calciphylaxis patients treated with STS and those who were not [38].

Bisphosphonates have also been used off-label for calciphylaxis management, similar to sodium thiosulfate and cinacalcet. Proposed mechanisms of action include hindering hydroxyapatite crystallization, diminishing macrophage activity, and lowering proinflammatory cytokines. By reducing calcium accumulation in arterial walls, bisphosphonates may inhibit vascular calcification. In a small prospective series, the incorporation of bisphosphonates was observed to slow down the progression of calciphylaxis in all cases within 2–4 weeks of initiating treatment. Additionally, significant enhancements in outcomes were noted compared to patients managed solely with supportive therapies such as debridement and low-calcium dialysate [6,38].

In these case-reports, the treatment approaches for calciphylaxis after PTx illustrated a comprehensive strategy that addresses both local and systemic patient care. These treatments included wound care often combined with antibiotics. The therapeutic arsenal included hyperbaric oxygen therapy [16] and STS [8,19,21]. To improve patients’ quality of life, pain management medications and nutritional support were also considered. Surgical interventions, such as debridement and even total mastectomy [18], or limb amputation [2] emphasize the severity of non-healing wounds and severe pain, underscoring the need for surgical solutions alongside medical treatments for serious complications. In some cases, stopping anti-vitamin K [22] and calcium supplements (our case), or adopting calcium-lowering strategies like low calcium dialysate, calcitonin agents, or bisphosphonates [17], demonstrated personalized care tailored to each patient’s situation for optimal outcomes. In our case, the patient, in addition to discontinuing calcium supplements, was treated with cinacalcet, resulting in a significant reduction in PTH levels. The patient did not receive bisphosphonates or sodium thiosulfate.

Limitations of this manuscript include the limited number of post-PTX calciphylaxis cases published in the literature, the heterogenous reporting of information such as pre-PTx PTH levels, calcium supplementation after PTx or other risk factors, and the absence of skin biopsy confirmation in some cases, including ours. Also, the late effect of severe persistent SHPT manifesting after PTx cannot be excluded. However, recognizing and understanding these cases is crucial for improving early diagnosis, guiding management strategies, and identifying potential risk factors to prevent post-PTx calciphylaxis.

## 5. Conclusions

The case presented highlights the challenges of managing SHPT and calciphylaxis developing after PTx in a dialysis patient. PTH fluctuations required intermittent cinacalcet therapy, eventually discontinued as biochemical stability was achieved. Long-term follow-up showed adequate calcium metabolism control with alfacalcidol, with no recurrence of ischemic lesions, emphasizing the need for individualized management.

It is imperative that clinicians must consider calciphylaxis in kidney disease patients with new painful skin lesions. While PTx represents one potential treatment option, it is important to recognize that calciphylaxis can still manifest postoperatively. A multidisciplinary, individualized approach is essential. Post-PTx hypocalcemia often occurs, so caution is needed with high-dose calcium supplements. Further studies are warranted to improve our understanding of this pathology and optimize treatment strategies.

## Figures and Tables

**Table 1:** BMD-DXA evaluations.

BMD-DXA	L1-L4 Z-Score	Femoral Neck Z-Score	Forearm Z-Score
2021	−1.4	−1.8	NA ^1^
2024	−1.3	−0.7	−5.8

BMD-DXA, bone mineral density-dual X-ray absorptiometry; ^1^ NA = not assessed.

**Table 2:** Reported cases of calciphylaxis following PTx.

Author	M/F	Age (y)	Type of RRT	HD/Pdvintage	PTx Indication	Pre-PTx PTH (pg/mL)	Type of PTx	Post-PTx PTH (pg/mL)	Distribution of the Lesions	Time After PTx	Diagnostic Methods	Risk Factors	Therapeutic Approach After PTx
**Poch et al., 1992 [15]**	M	62	HD	8 y	SHPT, severe signs of osteodystrophy	1205	Subtotal PTx	213	Distal lower and upper extremities	3 mo	Skin biopsy	Autoimmune conditions, prolonged application of topical therapies for psoriasis; deficiency of protein C; calcium supplementation for hypocalcemia	Calcitriol; calcium supplements; topical treatment for psoriatic lesions; transferred to PD 3 months later
**Oikawa et al., 2004 [16]**	M	32	PD-HD	13 y	SHPT	Unknown	PTx (not specified)	63 during the onset of symptoms	Back, lower abdominal wall, lower extremities, penile and scrotal involvement	2 y	Skin biopsy; X-ray; 99mTc-methylene disphosphonate; CT scan	Obesity followed by marked weight loss, corticosteroid medication	Hyperbaric oxygen for whole body; prostaglandin E1; continuous hemodiafiltration and endotoxin absorption; antibiotics; catecholamines; analgesics and narcotics
**Matstusoka et al., 2005 [17]**	F	57	HD	24 y	SHPT	Unknown	Total PTx with forearm autograft	120	Lower extremities	Unknown	Skin biopsy	Unknown	Medical and dermatological treatment
	M	42	HD	15 y	SHPT	Unknown	Total PTx with forearm autograft	705	Trunk and extremities	3 y	Skin biopsy	Unknown	Low calcium dialysate, calcitonin agent, bisphosphonates
	F	48	HD	16 y	SHPT	Unknown	Total PTx with forearm autograft	180	Lower extremities	Unknown	Skin biopsy	Unknown	Medical and dermatological treatment
**Bonilla et al., 2007 [18]**	F	59	HD	unknown	Calciphylaxis	Unknown	Subtotal PTx	Unknown	Breast	Unknown	Skin biopsy	Anticoagulation (specific type not mentioned)	Fine-needle aspiration; total mastectomy (because of the nonhealing wound and severe breast pain); another PTx
**Wahab et al., 2008 [13]**	M	33	HD	9 y	SHPT	3489	Subtotal PTx	47	Distal lower extremities	7 w	Skin biopsy	Unknown	Dermatological treatment, antibiotics, skin grafting
**Katikaneni et al., 2013 [19]**	F	62	HD	5 y	Calciphylaxis	1513	Subtotal PTx	406	Breast, thigh, and lower abdominal wall	16 mo	Skin biopsy	Obesity; DM2; calciphylaxis prior to PTx, calcium supplementation and vitamin D analog for hypocalcemia	25 g of STS 3 times weekly; high calcium dialysate, sevelamer carbonate
**Bashir et al., 2016 [20]**	M	46	HD	14 y	SHPT, Pathological fractures	2000	Subtotal PTx	891	Penile	2 w	CT scan	Calcium supplementation and vitamin D analog for hypocalcemia	Low calcium dialysate; high dose of sevelamer; local antibiotic; traditional treatment (honey, local herbs)
**Karmegam and Shetty, 2017 [21]**	M	60	HD	2.5 y	HPT and osteopenia	4191	Subtotal PTx	184	Lower extremity and buttocks	4 w	Skin biopsy	DM2, prednisone usage for pain relief	Wound care; antibiotics; increased duration of HD session; 25 g STS with all HD sessions; high-dose oral calcium and vitamin D supplements; oral opiods, topical lidocaine, gabapentine; prednisone
**Sanha et al., 2023 [8]**	F	26	HD	4 y	Brown tumor due to SHPT	>2000	Total PTx with forearm autograft	43 (on the first postoperative day), 118 (4 weeks later)	Distal lower extremities	3 w	Clinical diagnosis (negative early biopsy)	No risk factors mentioned	Wound care; pain management; antibiotics, STS
**Hristov et al., 2023 [22]**	M	46	HD	8 y	SHPT	>1400	Subtotal PTx	values of 120–250	Unknown	Unknown	Unknown	Unknown	Surgical debridement; antibiotics; discontinuation of anti-vitamin K; nutritional support
	M	49	HD	11 y	SHPT	>1400	Subtotal PTx	values of 120–250	Unknown	Unknown	Unknown	Unknown	Surgical debridment; antibiotics; discontinuation of anti-vitamin K; nutritional support
**Smith et al., 2023 [2]**	F	60	KTx	7 y	SHPT	396	Total PTx	<9	Left lower leg	3 y	Histopathology	DM, anticoagulation with anti-vitamin K	Limb amputation; STS, discotinuation of anti-vitamin K; vitamin K2 supplementation
**Our study, 2024**	M	36	HD-DP-HD	16 y	SHPT	2100	Total PTx	330	Distal upper extremities	8 w	Clinical diagnosis	Calcium supplementation and vitamin D analog for hypocalcemia	Discontinuation of calcium supplementation

M, male; y, years; RRT, renal replacement therapy; mo, months; F, female; HD, hemodialysis; PD, peritoneal dialysis; SHPT, secondary hyperparathyroidism; PTx, parathyroidectomy; DM2, diabetes mellitus type 2; w, weeks; STS, sodium thiosulfate; KTx, kidney transplantation.

## Data Availability

The original contributions presented in the study are included in the article. Further inquiries can be directed to the corresponding author.

## Abbreviations

The following abbreviations are used in this manuscript:

CUA	calcific uremic arteriopathy
CKD	chronic kidney disease
KTR	kidney transplant recipient
HD	hemodialysis
PTx	parathyroidectomy
PTH	parathormone
Ca	calcium
P	phosphate
MGP	matrix G1a protein
ESRD	end-stage renal disease
RRT	renal replacement therapy
CKD-MBD	chronic kidney disease-mineral bone disorder
DXA	dual X-ray absorptiometry
BMD-DXA	bone mineral density-dual X-ray absorptiometry
BMP	bone morphogenetic protein
iPTH	intact parathormone
DM	diabetes mellitus
SHPT	secondary hyperparathyroidism
STS	sodium thiosulfate
NKF-KDOQI	National Kidney Foundation-Kidney Disease Outcomes Quality Initiative
